# Supplementary material for: On the use of random graphs in analysing resource utilization in urban systems
Source: R Soc Open Sci. 2020 Apr 15;7(4):200087. doi: 10.1098/rsos.200087 (PMC7211871; doi:10.1098/rsos.200087)
Supplement: Supplementary Material and Methods [file rsos200087supp1.pdf]

ROYAL SOCIETY
OPEN SCIENCE

On the Use of Random Graphs in Analysing Resource Utilisation in Urban Systems: Supplementary Material

Hadi Arbabi[1,*], Giuliano Punzo[2], Gregory Meyers[1], Ling Min Tan[1], Qianqian Li[1], Danielle Densley Tingley[1], Martin Mayfield[1]

1 Department of Civil & Structural Engineering, University of Sheffield, Sheffield S1 3JD, UK
2 Department of Automatic Control & Systems Engineering, University of Sheffield, Sheffield S1 3JD, UK

1. APPENDIX A – Material and Method

In this section we outline the Monte Carlo protocol used throughout and for the numerical validation of Proposition 1 (see Data Accessibility for the Python code used and data generated).

Monte Carlo algorithm

SI Algorithm 1 shows the basic Monte Carlo method used to produce the numerical data sets used in this paper. In this algorithm, a single Monte Carlo sample of single directed network is generated with the amount of flow imported and utilised by the network is calculated. While the size of the network is predetermined by the user through setting the number of vertices and edges, the network topology, vertex efficiencies and edge flows are randomly drawn. For the algorithm, variable and object names are in lowercase with underscores to separate words whilst functions and object methods use the NerdCaps naming convention (*1*).

Line 1 of the algorithm shows where a random network with a specified number of vertices and edges is instantiated. This is followed by the $lambdas$, $phis$, and $flows$ being randomly drawn and assigned to the vertices and edges of the network on lines 2 through to 7. The function `DrawRandomUniformValues(`$size$`)` returns a vector of floats determined by the passed parameter $size$ and are each independently drawn from a uniform distribution of values in (0, 1).

On line 11 a for loop is used to cycle through each of the vertices of the network. The $network$ method `GetInEgdesOfNode()` on line 15 returns a list of the directed edges going to network vertex n. These edges are then cycled through by in another loop on line 16 so that

i. the amount of flow into vertex n is accumulated by the variable $flow\_in$ on line 18,
ii. the amount of flow utilised by the network is calculated on a per edge basis and accumulated by the variable $flow\_utilised$ on line 21.

The $network$ method `GetOutEgdesOfNode()` acts similar to `GetInEgdesOfNode()` but returns a list of the directed edges leaving the network vertex n instead. Like the preceding for loop, the out edges are cycled through so that the flow out of vertex n can be accumulated in the variable $flow\_out$ on line 27. On line 29, the accumulated flow going into vertex n is subtracted from accumulated flow leaving vertex n to check if the vertex requires additional imported flow to meet the out flow demand of vertex n enforcing the equilibrium conditions. If there is a deficit, the imported flow is recorded and accumulated for the whole network by the variable $flow\_imported$ on line 30.

Finally, once the Monte Carlo simulation has finished, the effectiveness of the network instance, i.e. a single Monte Carlo sample, can be calculated by dividing the sample's $flow\_utilised$ by $flow\_imported$. This is following the mathematical formulation given in the main text, Equation 3. SI Table 1 shows a summary

*Author for correspondence (h.arbabi@sheffield.ac.uk).

SI Algorithm 1. Monte Carlo Implementation

```
Require: number_of_nodes ≥ 2
Require: number_of_edges ≥ 1
network ← CreateDirectedRandomNetwork(number_of_nodes, number_of_edges)
lambdas ← DrawRandomUniformValues(size = number_of_nodes)
phis ← DrawRandomUniformValues(size = number_of_nodes)
flows ← DrawRandomUniformValues(size = number_of_edges)
network.SetNodeAttributes(lambdas)
network.SetNodeAttributes(phis)
network.SetEgdeAttributes(flows)

flow_imported ← 0
flow_utilised ← 0
for n ← 0 to number_of_nodes do
    flow_in ← 0
    flow_out ← 0

    in_edges ← network.GetInEgdesOfNode(n)
    for each edge in in_edges do
        flow ← GetFlowValue(edge)
        flow_in ← flow_in + flow
        node ← GetSourceNode(edge)
        flow_eff ← (1 − node.lambda)/(node.lambda ∗ node.phi)
        flow_utilised ← flow_utilised + flow ∗ flow_eff

    out_edges ← network.GetOutEgdesOfNode(n)
    for each edge in out_edges do
        flow ← GetFlowValue(edge)
        node ← GetSourceNode(edge)
        flow_out ← flow_out + flow/(node.lambda ∗ node.phi)

    if flow_out − flow_in > 0 then
        flow_imported ← flow_imported + flow_out − flow_in
```

description of the numerical runs including vertex and simulation counts included in the main text and in this supplementary material.

Numerical Validation of Proposition 1

To show that the Monte Carlo simulations match up to their analytical counterpart, a Monte Carlo simulation was run for the tractable case of the upper-limit boundary for effectiveness in fully-connected networks with identical vertices and edges, Proposition 1. To align with this case, SI Algorithm 1 and the accompanying Python script can be modified such that

i. all network flows are assigned the same randomly drawn value,
ii. every ϕ in a network is assigned the same randomly drawn value, and also
iii. every λ in a network is assigned the same randomly drawn value.

SI Table 1. Summary of the Monte Carlo simulations and their setting

	**MC Set 1**	**MC Set 2**	**MC Set 3**
MC Count	1M	50K	50K
Vertex Range	N in range(2,30,1)	N in range(2,30,1)	N in range(2,30,1)<br>N in range(30, 100, 10)<br>N in range(100,1000,100)<br>N in range(1000,5000,1000)
Edge Range	$E = N \times (N-1)$	E in range(1, $N \times (N-1)$,1)	$E = N \times (N-1)$
Vertex Parameters	$\forall i,j \begin{cases} \lambda_i = \lambda_j = \lambda \sim U(0,1), \\ \phi_i = \phi_j = \phi \sim U(0,1), \\ F_i = F_j = F \sim U(0,1) \end{cases}$	$\forall i \begin{cases} \lambda_i \sim U(0,1), \\ \phi_i \sim U(0,1), \\ F_i \sim U(0,1) \end{cases}$	$\forall i \begin{cases} \lambda_i \sim U(0,1), \\ \phi_i \sim U(0,1), \\ F_i \sim U(0,1) \end{cases}$

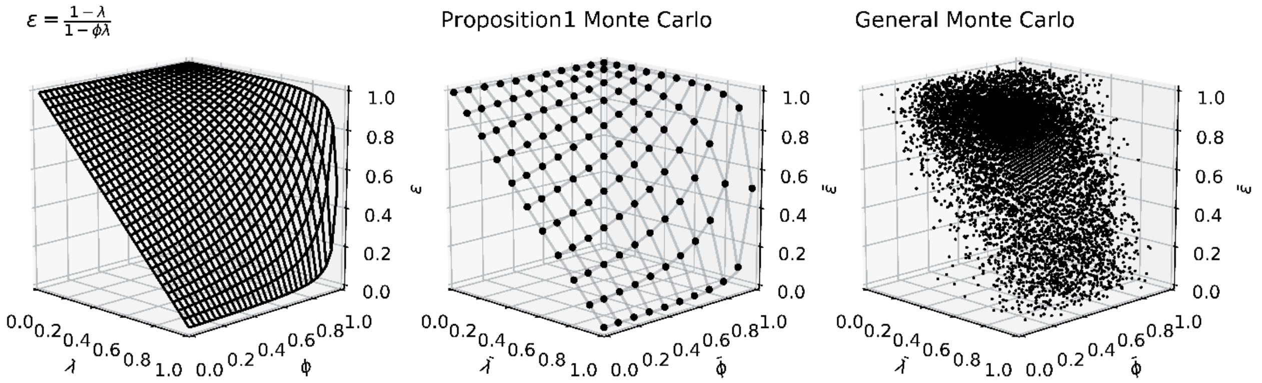

SI Figure 1. Left: theoretical upper-limit of the effectiveness of resource utilisation for a homogeneous system (main manuscript Proposition 1). Middle: Monte Carlo simulation of Proposition 1 for networks with vertex count between 2 to 30 averaged over 1M runs each shown over the theoretical surface. Right: general Monte Carlo simulations for networks with vertex count between 2 to 30 averaged over 50K runs each.

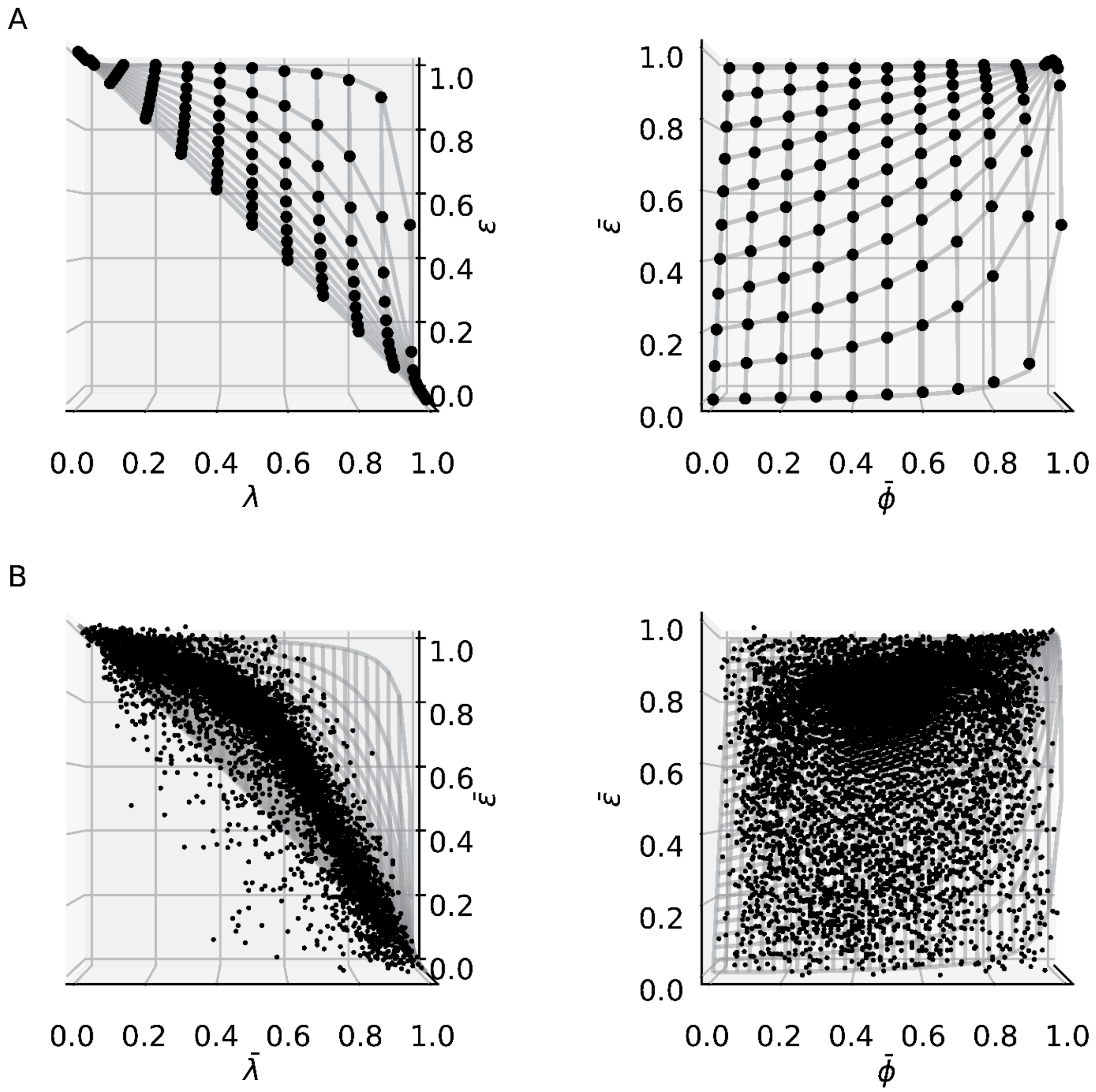

SI Figure 2. Variations of Monte Carlo estimated effectiveness, ϵ, against efficiency factors λ and ϕ for the upper-limit of homogeneous networks (A) and for the general case (B) – note that the grey wireframe shows analytical upper-limit according to $\epsilon = \frac{1-\lambda}{1-\phi\lambda}$.

2. APPENDIX B – Monte Carlo effectiveness distributions

In this appendix we provide supplementary figures for the Beta approximation of the effectiveness of resource utilization documenting the effectiveness distribution as a function of systems' size, i.e. number of vertices, and connectivity, i.e. number of edges.

The fully connected network effectiveness distributions shown can be approximately fitted with a Beta distribution. SI Figures 3 and 4 plot the α and β shape parameters and their means over vertex and edge count respectively. Note that each data point corresponds with estimates for 50K simulation runs for a given vertex and edge count. SI Figures 5 to 7 show the empirical distributions of ϵ for given vertex and edge counts overlaid with their approximated Beta fit.

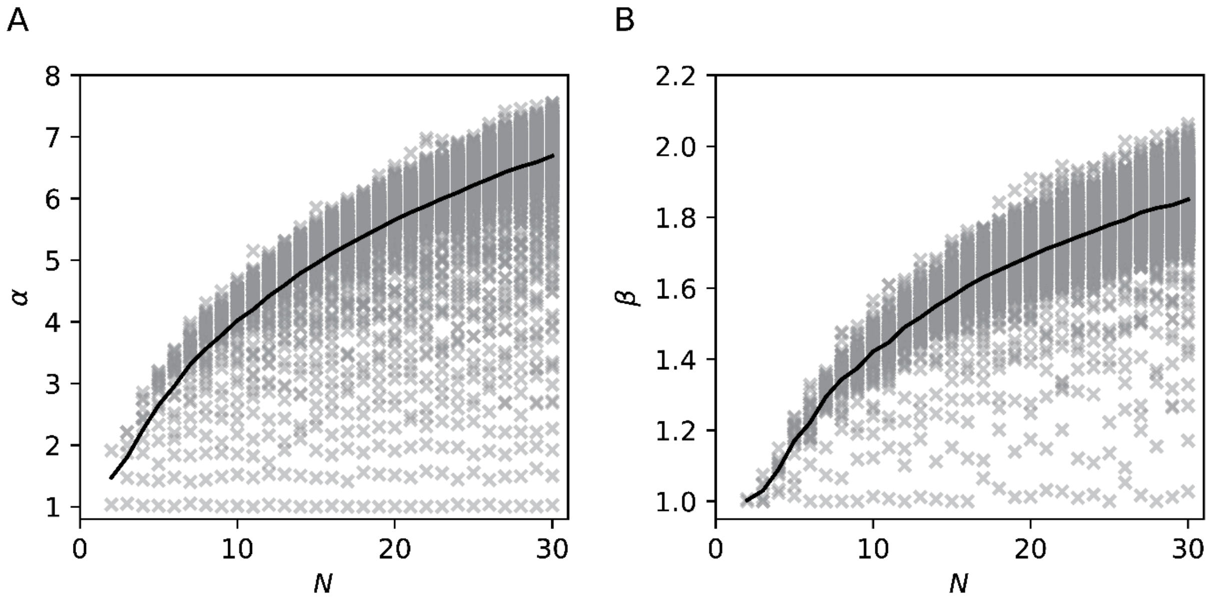

SI Figure 3. Estimated Beta shape factors and their means for networks with various vertex count, N.

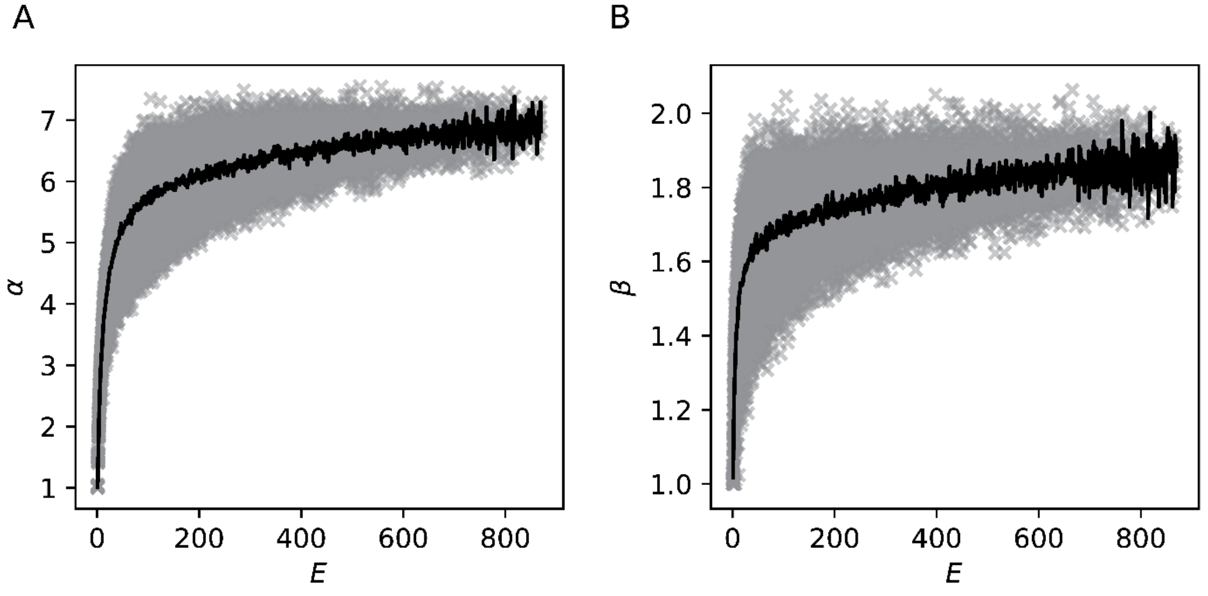

SI Figure 4. Estimated Beta shape factors and their means for networks with various edge count, E – note that the increased fluctuations in means for higher edge counts is a results of fewer MC samples as higher edge counts occur in fewer networks due to minimum vertex count required.

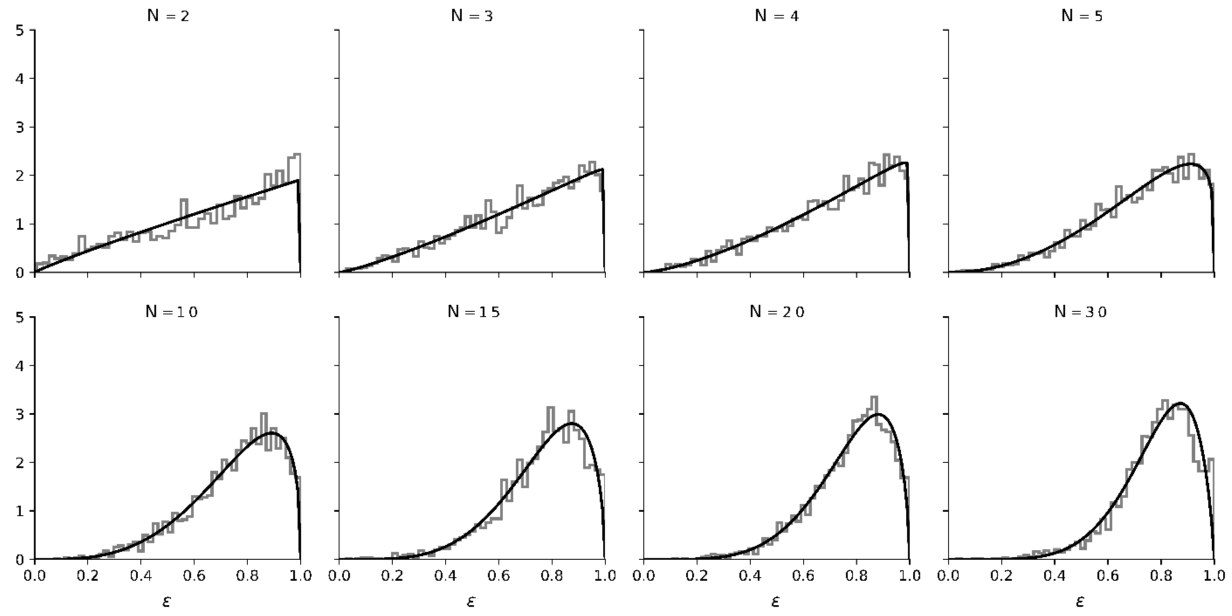

SI Figure 5. Empirical distributions of ϵ for fully-connected networks of various vertex count fitted with their approximated Beta distribution.

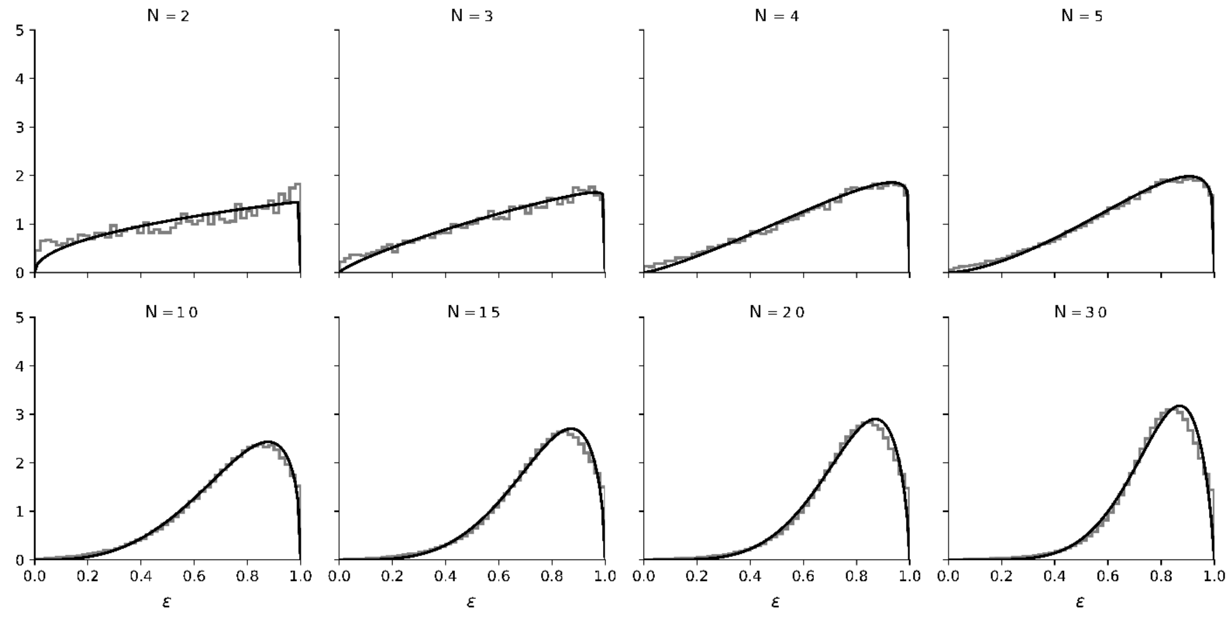

SI Figure 6. Empirical distributions of ϵ for all networks of a given vertex count overlaid with the Beta distributions with shape factors averaged across all estimated for a given vertex count, SI Figure 3.

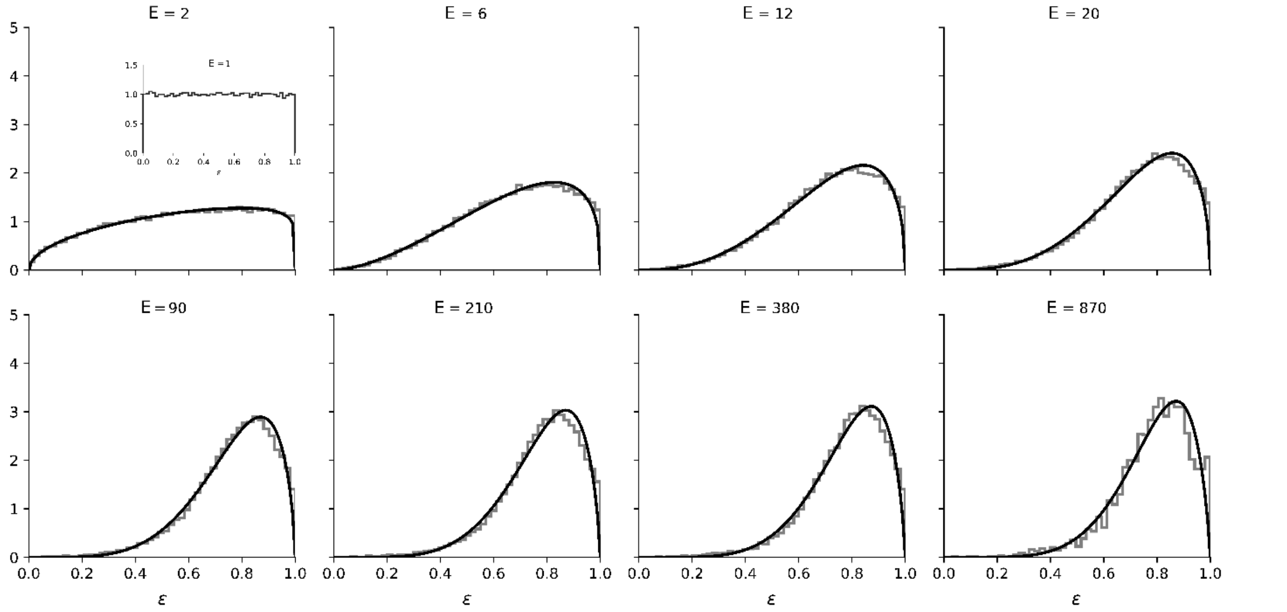

SI Figure 7. Empirical distributions of ϵ for all networks of a given edge count overlaid with the Beta distributions with shape factors averaged across all estimated for a given edge count, SI Figure 4 – inset shows empirical distribution of ϵ from all networks with exactly one edge.

Funding Statement
G.P. time was supported by EPSRC Engineering Complexity Resilience Network Plus [EP/N010019/1]. G.M. time was supported by EPSRC City Observatory Research Platform for Innovation and Analytics [EP/R013411/1]. L.M.T. acknowledges support from the Grantham Centre for Sustainable Futures at the University of Sheffield. Q.L. acknowledges support from Sheffield Urban Flows Observatory.

Data Accessibility
The Python script used for the creation and analysis of the Monte Carlo simulations is available at (https://github.com/cip15ha/randomgraph-resource-utilisation) and has been uploaded as part of the Supplementary Material. The simulation data used for the figures is available from the authors upon request.

Authors' Contributions
H.A. and G.M. conceived the study; G.M., H.A., and L.M.T. undertook the numerical study; G.P., Q.L., and H.A. undertook the analytical study; H.A., D.D.T., and M.M. contributed to the discussion; H.A., G.P., G.M. and L.M.T undertook the writing; H.A. assembled the manuscript. All authors gave final approval for publication and agree to be held accountable for the work performed therein.

References

1. B. Hayes, The Semicolon Wars. *Am. Sci.* **94** (2006), pp. 299–303.
